# Predictive Value of Multiparametric Magnetic Resonance Imaging (T2-weighted Imaging and Apparent Diffusion Coefficient) for Pathological Grading of Prostate Cancer: a Meta-Analysis

**DOI:** 10.1590/S1677-5538.IBJU.2024.0509

**Published:** 2025-02-10

**Authors:** Subo Zhang, Jinxin Wan, Yongjun Xu, Leiming Huo, Lei Xu, Jiabao Xia, Zhitao Zhu, Jingfang Liu, Yan Zhao

**Affiliations:** 1 The Second People's Hospital of Lianyungang Department of Medical Imaging China Department of Medical Imaging, The Second People's Hospital of Lianyungang, Jiangsu Province, China; 2 Lianyungang Clinical College Jiangsu University Department of Medical Imaging Lianyungang City China Department of Medical Imaging, Lianyungang Clinical College Jiangsu University, Lianyungang City, Jiangsu Province, China; 3 The Second People's Hospital of Lianyungang Affiliated with Kangda College of Nanjing Medical University Department of Medical Imaging Lianyungang China Department of Medical Imaging, The Second People's Hospital of Lianyungang Affiliated with Kangda College of Nanjing Medical University, Lianyungang City, Jiangsu Province, China; 4 The Second People's Hospital of Lianyungang Department of Respiratory Lianyungang China Department of Respiratory, The Second People's Hospital of Lianyungang, Lianyungang City, Jiangsu Province, China

**Keywords:** Prostatic Neoplasms, Multiparametric Magnetic Resonance Imaging, Meta-Analysis [Publication Type]

## Abstract

**Objective:**

This meta-analysis aimed to evaluate the predictive value of multiparametric magnetic resonance imaging (mpMRI), specifically T2-weighted imaging (T2WI) and apparent diffusion coefficient (ADC) maps, in the pathological grading of prostate cancer.

**Methods:**

A comprehensive literature search was conducted across multiple databases, including PubMed, the China National Knowledge Infrastructure dataset, Web of Science, Springer Link and Cochrane Library. Studies evaluating the use of mpMRI for prostate cancer grading were included. The quality of the included studies was assessed using the risk of bias tool. Meta-analyses were performed to calculate pooled areas under the curve (AUC) and prostate cancer detection rates.

**Results:**

Seven studies met the inclusion criteria, comprising 843 patients in the experimental group and 962 in the control group. The meta-analysis revealed a significant improvement in diagnostic performance with mpMRI, with a pooled mean difference in AUC of 0.10 (95% confidence interval [CI]: 0.04–0.16, p = 0.002) favouring the mpMRI group. The odds ratio for prostate cancer detection was 2.60 (95% CI: 1.57–4.29, p = 0.0002), indicating a higher detection rate with mpMRI compared with standard techniques. Substantial heterogeneity was observed among the studies (I² = 73% for AUC and 66% for detection rate).

**Conclusion:**

This meta-analysis demonstrates that mpMRI, particularly T2WI and ADC imaging, has a significant predictive value in the pathological grading of prostate cancer. The technique shows improved diagnostic accuracy and higher cancer detection rates compared with conventional methods. However, the substantial heterogeneity among studies suggests that standardisation of mpMRI protocols and interpretation criteria is needed.

## INTRODUCTION

Prostate cancer remains one of the most prevalent malignancies affecting men worldwide, with significant implications for public health (1). The accurate diagnosis and grading of prostate cancer are crucial for determining appropriate treatment strategies and predicting patient outcomes. Traditionally, prostate-specific antigen (PSA) testing and systematic transrectal ultrasound (TRUS)-guided biopsies have been the standard approach for prostate cancer detection and grading. However, these methods have limitations, including overdiagnosis of clinically insignificant cancers and undersampling of significant tumours (2).

In recent years, multiparametric magnetic resonance imaging (mpMRI) has emerged as a promising tool in the diagnostic armamentarium for prostate cancer. Multiparametric magnetic resonance imaging (MRI) combines anatomical T1-weighted and T2-weighted imaging (T2WI) with functional techniques, such as diffusion-weighted imaging (DWI) and dynamic contrast-enhanced (DCE) imaging (3). Among these, T2WI provides excellent soft-tissue contrast and anatomical detail, while apparent diffusion coefficient (ADC) maps derived from DWI offer insights into tissue cellularity and tumour aggressiveness (4).

The potential of mpMRI to improve prostate cancer detection and characterisation has led to its increasing adoption in clinical practice. The Prostate Imaging Reporting and Data System (PI-RADS) has been developed to standardise the acquisition, interpretation and reporting of prostate mpMRI (5). However, the precise role of mpMRI in predicting the pathological grade of prostate cancer remains a subject of ongoing research and debate.

Several studies have investigated the correlation between mpMRI parameters, particularly T2WI and ADC values, and prostate cancer Gleason scores (6, 7). These studies suggest that mpMRI can provide valuable information for distinguishing between low- and high-grade prostate cancers. However, the results have been heterogeneous, and the overall predictive value of mpMRI for pathological grading remains unclear.

Multiparametric MRI, specifically T2WI and ADC maps, has significant predictive value in the pathological grading of prostate cancer, offering improved diagnostic accuracy and higher cancer detection rates compared with conventional diagnostic methods. This meta-analysis aims to evaluate comprehensively the predictive value of mpMRI, specifically focusing on T2WI and ADC imaging, in the pathological grading of prostate cancer. By synthesising data from multiple studies, we seek to provide a more robust assessment of the diagnostic performance of mpMRI and its potential role in clinical decision-making for prostate cancer management.

## METHODS

### Search strategy and study selection

A comprehensive literature search was conducted across multiple electronic databases, including PubMed, the China National Knowledge Infrastructure (CNKI), Web of Science, Springer Link and Cochrane Library. The search strategy employed a combination of Medical Subject Headings terms and key words related to magnetic resonance imaging and prostate cancer. The specific search terms included variations of ‘Magnetic Resonance Imaging’, ‘NMR Imaging’, ‘Zeugmatography’, ‘fMRI’, ‘Functional Magnetic Resonance Imaging’, ‘MRI Scans’, ‘Spin Echo Imaging’ and ‘Magnetic Resonance Image’ for the imaging modality. These were combined with terms related to prostate cancer, including ‘Neoplasms, Prostatic’, ‘Prostate Cancer’ and ‘Cancer of Prostate’. This study has been registered at inplasy.com, registration number is INPLASY202520044.

The initial database search identified a total of 214 records: 16 from PubMed, 107 from CNKI, 17 from Web of Science, 8 from Springer Link and 66 from Cochrane Library. After removing duplicates, 107 unique records remained for screening.

### Inclusion and exclusion criteria

This systematic review employed specific inclusion and exclusion criteria to ensure the relevance and quality of the included studies. Eligible studies evaluated the use of mpMRI (including T2WI and ADC) for prostate cancer detection or grading, included a comparison group, reported outcomes in terms of areas under the curve (AUC), sensitivity, specificity or prostate cancer detection rates, involved human subjects and were published in English or Chinese. Studies were excluded if they were case reports, reviews or conference abstracts, focused solely on other imaging modalities or MRI sequences, lacked a clear comparison group or provided insufficient data for quantitative analysis.

### Study selection process

The study selection process followed a systematic approach. Initially, 89 records were excluded based on title screening. The remaining 18 records underwent abstract review, resulting in the exclusion of 10 more studies. A full-text assessment was performed on 8 articles, of which 1 was excluded due to non-synthesisable results. Ultimately, 7 studies were included in the qualitative and quantitative synthesis.

### Data extraction

Data extraction was performed by two independent reviewers using a standardised form. The extracted information encompassed key details, such as author names and publication year, MRI parameters used, sample sizes for both intervention and control groups, age range of participants, outcome measures and study design. This comprehensive data extraction process allowed for a thorough analysis of the included studies and facilitated the synthesis of results across different research efforts in the field of multiparametric MRI for prostate cancer detection and grading.

### Quality assessment

The risk of bias in the included studies was assessed using the Cochrane Collaboration's tool for assessing risk of bias in randomised trials. This tool evaluates seven domains: random sequence generation, allocation concealment, blinding of participants and personnel, blinding of outcome assessment, incomplete outcome data, selective reporting and other biases. Each domain was categorised as low risk, unclear risk or high risk of bias.

## Statistical analysis

Meta-analyses were performed using Review Manager 5.3 software. For continuous outcomes (AUC), the mean difference with 95% confidence interval (CI) was calculated. For dichotomous outcomes (prostate cancer detection rates), odds ratios (ORs) with 95% CI were computed.

The inverse variance method with random-effects models was used to account for potential heterogeneity among studies. Heterogeneity was assessed using the I² statistic, with I² values of 25%, 50% and 75% considered as low, moderate and high heterogeneity, respectively. Forest plots were generated to represent the results of the meta-analyses visually. Funnel plots were created to assess potential publication bias. All statistical tests were two-sided, with a p-value of <0.05 considered statistically significant.

## RESULTS

### Study characteristics

**Figure 1:**
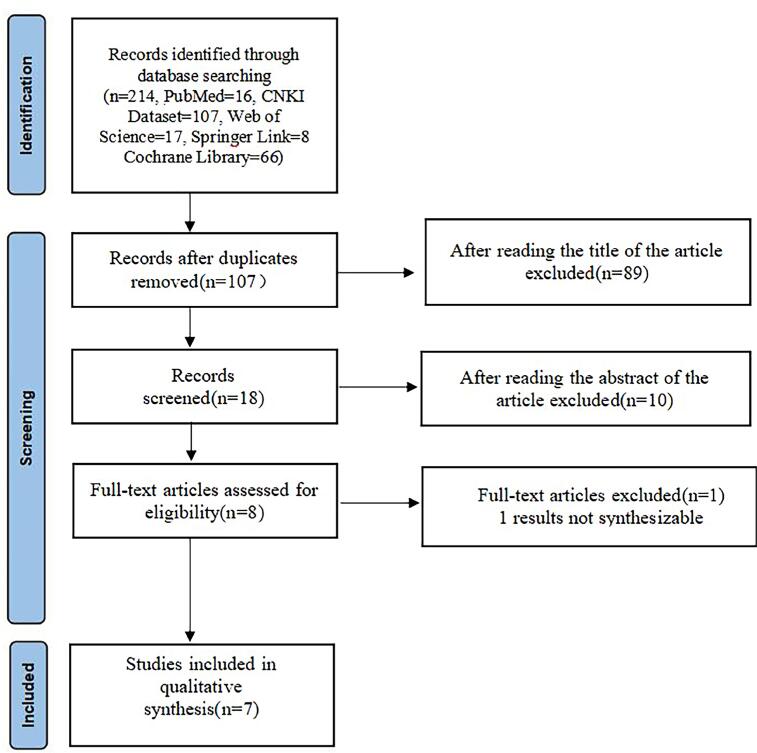
The flow chart of literature screening.

The systematic review process resulted in the inclusion of seven studies (8–14) for qualitative and quantitative synthesis. These studies, published between 2007 and 2024, collectively involved 843 patients in the experimental group (mpMRI) and 962 patients in the control group (Figure-1).

The basic characteristics of the included studies are summarised in Table-1. The included studies utilised various MRI parameters, with all studies incorporating T2WI and ADC maps. Some studies also included additional parameters, such as DCE imaging and DWI. The age range of participants across studies was 26–91 years, with most studies focusing on men in their 60s and 70s.

**Table 1:** The summarized basic characteristics of included studies.

Author (year)	MRI	Number	Invention condition	Age	Outcome index	Research type	Reference
		invention/control					
Zhang, et al. 2024	T2WI, DWI, ADC	106/105	MRI	-	AUC	Controlled trial	(8)
Salami, et al. 2017	T2WI, DWI, ADC	202/110	Mp-MRI	59-72	AUC	Controlled trial	(9)
Morgan, et al. 2007	T2WI, DWI	27/27	DW-MRI	-	Sensitivity and specificity for tumor identification, AUC	Controlled trial	(10)
Wu, et al. 2019	K_trans_ K_ep_, ADC	17/22	Mp-MRI	60-79	AUC	Controlled trial	(11)
Kasivisvanathan, et al. 2018	T2WI	252/248	MRI	-	Sensitivity and specificity for tumor	Controlled trial	(12)
Wang, et al. 2015	DWI, MPS	132/345	Mp-MRI	**26-91**	PI-RADS score, AUC, accuracy	Controlled trial	**(** **13** **)**
Porpiglia, et al. 2017	ADC, DWI	107/105	Mp-MRI	-	AUC, accuracy	Controlled trial	(14)

MP-MRl = multiparametric magnetic resonance imaging; Pl-RADS = Prostate lmaging Reporting and DataSystem; AUC = areas under the curve; ADC = apparent diffusion coefficient; DWI = diffusion-weighted imaging; T2WI = T2-weighted imaging; MPS = Multiphasic Screening

### Quality assessment

**Figure 2:**
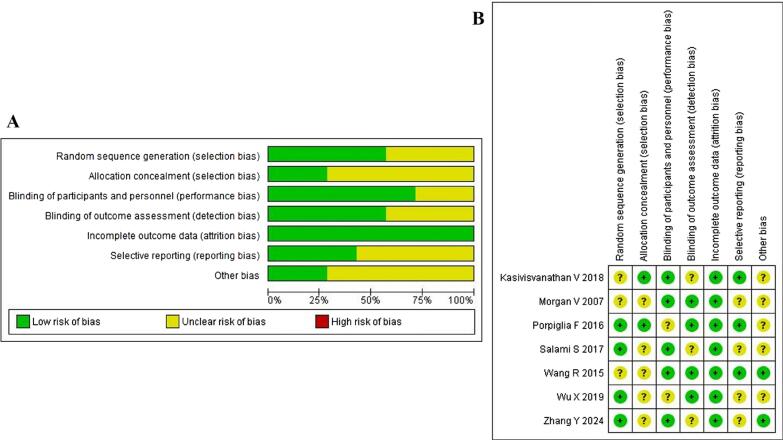
Risk of bias graph. A: Review authors' judgements about each risk of bias item presented as percentages across all included studies. B: Review authors' judgements about each risk of bias item for each included study.

The risk of bias assessment for the included studies, as presented in Figure-2, reveals a moderate to high overall quality, though with some areas of concern. Random sequence generation was generally well-handled, with approximately 60% of studies judged to have a low risk of bias, while the remaining 40% had an unclear risk. Allocation concealment was less clearly reported, with only 30% of studies demonstrating a low risk of bias and 70% having an unclear risk. Blinding of participants and personnel was adequately addressed in 75% of studies, showing a low risk of bias, while 25% were unclear. The blinding of outcome assessment was evenly split, with half of the studies having a low risk and half having an unclear risk. Notably, all studies (100%) were assessed as having a low risk of bias regarding incomplete outcome data, indicating strong reporting in this domain. Selective reporting was a concern in 60% of studies with an unclear risk, while 40% demonstrated a low risk. Last, other potential sources of bias were largely unclear, with 70% of studies having an unclear risk and only 30% judged to have a low risk. These findings highlight areas where future research could improve methodological clarity and reporting, particularly in allocation concealment, selective reporting and addressing other potential sources of bias.

### Diagnostic accuracy

**Figure 3:**
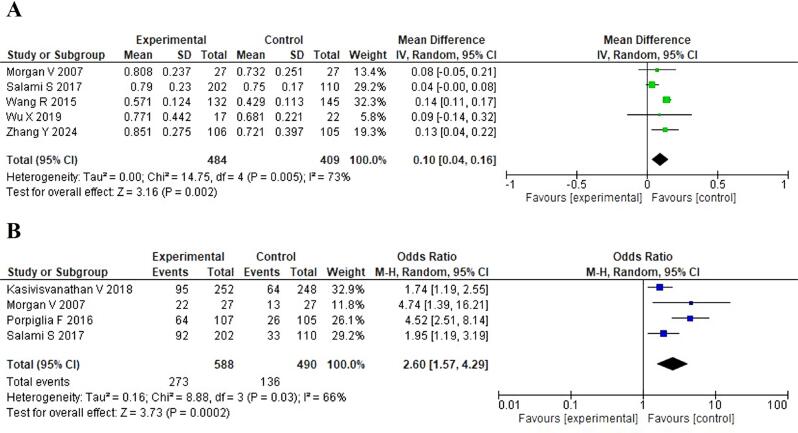
Diagnostic accuracy and prostate cancer detection rate. A) Forest map of area under the curve (AUC). B) Forest map of the rate of prostate cancer detection.

The meta-analysis of the AUC included five studies with a total of 484 patients in the experimental group and 409 in the control group. The forest plot (Figure-3A) demonstrates a significant improvement in diagnostic performance with mpMRI. The pooled mean difference in AUC was 0.10 (95% CI: 0.04–0.16) favouring the mpMRI group. This result was statistically significant (Z = 3.16, p = 0.002). However, substantial heterogeneity was observed among the studies (I² = 73%), indicating considerable variability in the reported AUC values across different studies.

### Prostate cancer detection rate

Four studies reported on the rate of prostate cancer detection, involving a total of 588 patients in the experimental group and 490 in the control group. The forest plot (Figure-3B) shows that mpMRI significantly improved the detection rate of prostate cancer. The pooled OR was 2.60 (95% CI: 1.57–4.29) in favour of mpMRI. This result was highly statistically significant (Z = 3.73, p = 0.0002).

Similar to the AUC analysis, substantial heterogeneity was observed among these studies (I² = 66%), suggesting variability in the detection rates across different study populations and settings.

### Publication bias

**Figure 4:**
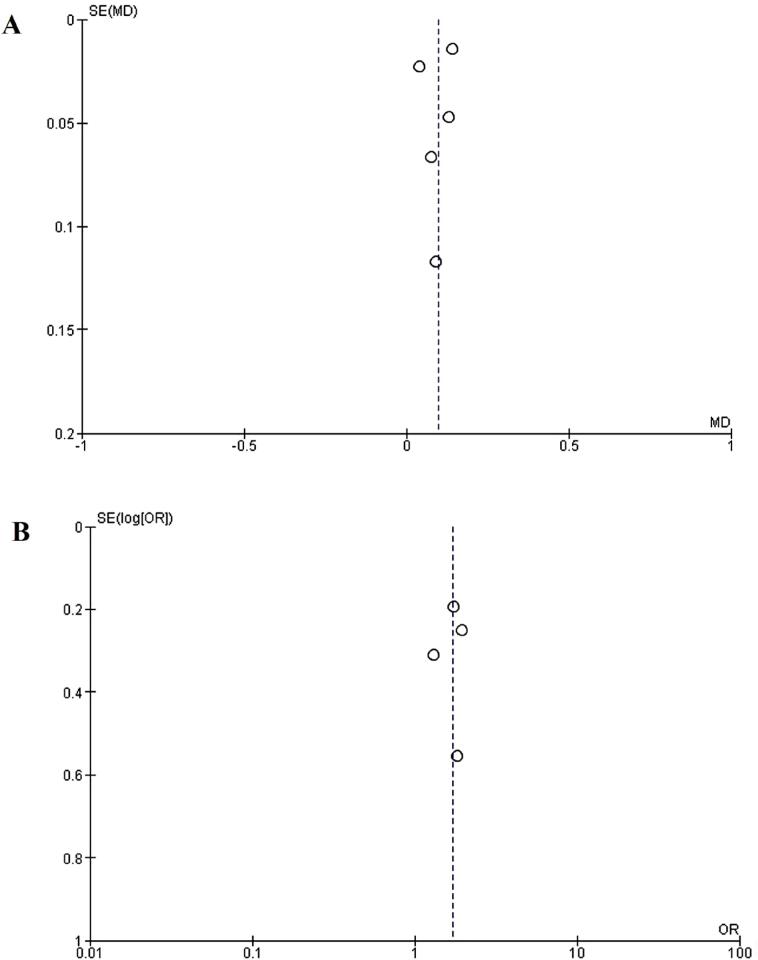
Publication Bias. A) Funnel map of area under the curve (AUC). B) Funnel map of the rate of prostate cancer detection.

Funnel plots for both the AUC (Figure-4A) and prostate cancer detection rate (Figure-4B) analyses were generated to assess potential publication bias. Although the limited number of included studies makes it challenging to draw definitive conclusions about publication bias, the funnel plots do not show clear evidence of asymmetry, suggesting that significant publication bias is unlikely.

## DISCUSSION

This meta-analysis provides a comprehensive evaluation of the predictive value of mpMRI, specifically focusing on T2WI and ADC maps, in the pathological grading of prostate cancer. The results demonstrate significant improvements in both diagnostic accuracy and prostate cancer detection rates when using mpMRI compared with conventional diagnostic methods.

For the diagnostic accuracy, the pooled analysis of AUC values revealed a mean difference of 0.10 (95% CI: 0.04–0.16) favouring mpMRI. This finding suggests that mpMRI offers superior diagnostic performance in distinguishing different grades of prostate cancer. The improved accuracy can be attributed to the combination of anatomical information from T2WI and functional data from ADC maps, which together provide a more comprehensive assessment of prostate tissue characteristics (15).

The enhanced diagnostic accuracy of mpMRI has important clinical implications. It may allow for more precise targeting of biopsies, potentially reducing the number of unnecessary procedures and improving the detection of clinically significant cancers. Furthermore, accurate grading is crucial for treatment planning, as it influences decisions regarding active surveillance, focal therapy or radical treatment options (16).

Our meta-analysis demonstrated that mpMRI significantly improved the detection rate of prostate cancer, with a pooled OR of 2.60 (95% CI: 1.57–4.29). This finding aligns with previous studies suggesting that mpMRI can detect prostate cancers that may be missed by conventional systematic biopsies (17). The improved detection rate is especially important for identifying clinically significant cancers while potentially reducing overdiagnosis of indolent tumours.

The higher detection rate with mpMRI may be explained by its ability to visualise suspicious areas within the prostate that can be targeted for biopsy. This targeted approach contrasts with the systematic sampling used in conventional TRUS-guided biopsies, which may miss cancers in areas not routinely sampled (18).

Despite the promising results, it is important to note the substantial heterogeneity observed among the included studies (I² = 73% for AUC and 66% for detection rate). This heterogeneity may be attributed to several factors. Variability in MRI protocols is a key consideration; while all studies included T2WI and ADC, some incorporated additional sequences, such as DCE imaging, which may influence diagnostic performance (19). Differences in study populations also contribute to this heterogeneity, as the age ranges and risk profiles of participants varied across studies, potentially affecting the prevalence and characteristics of detected cancers (20). Furthermore, variations in reference standards, particularly in the methods used for pathological confirmation and grading, may have differed between studies, introducing potential bias (21).

The level of interpreter experience is another crucial factor. The expertise in reading prostate mpMRI can significantly impact diagnostic accuracy and may have varied across studies (22). This variability in reader experience could contribute substantially to the observed differences in results. Last, it is important to consider technological advancements over time. The included studies span nearly two decades, during which MRI technology and interpretation techniques have evolved considerably (23). This temporal factor adds another layer of complexity to the heterogeneity observed in the meta-analysis. These sources of heterogeneity highlight the need for standardisation in mpMRI acquisition, interpretation and reporting. Initiatives such as PI-RADS aim to address this issue, but further refinement and widespread adoption are necessary to improve consistency across different clinical settings (24). Morote et al. reported (25) that the Barcelona MRI predictive model has been successfully validated when mpMRI was reported with PI-RADS v2.1 and prostate biopsies were conducted via the transrectal and transperineal route. Lv et al. found (26) that the mean PSA density combined with PI-RADS showed utility in guiding optimisation of the prostate biopsy mode. Higher PSAD and PI-RADS values were associated with greater confidence in implementing mono-targeted biopsy and safely omitting systematic biopsy, thus effectively balancing the benefits and risks. In addition, some relevant reports have investigated the accuracy and key role of mpMRI in predicting different prostate cancers (27, 28).

The limited number of studies meeting our inclusion criteria precluded subgroup analyses that may have shed light on the impact of specific factors on diagnostic performance. Additionally, the lack of individual patient data restricted our ability to assess the influence of patient characteristics on mpMRI performance. However, the findings of this meta-analysis support the integration of mpMRI into the diagnostic workflow for prostate cancer. The improved diagnostic accuracy and detection rates suggest that mpMRI could play a crucial role in reducing unnecessary biopsies, improving the detection of clinically significant cancers and guiding treatment decisions (12).

Future research should focus on large-scale prospective studies with standardised protocols to further elucidate the role of mpMRI in prostate cancer management. However, several challenges need to be addressed to optimise the clinical utility of mpMRI in prostate cancer management. Standardisation remains a crucial issue, with ongoing efforts required to harmonise mpMRI protocols, interpretation criteria and reporting systems to reduce variability in clinical practice (29). Additionally, exploring the integration of mpMRI with novel biomarkers may further enhance its predictive value in prostate cancer grading and risk stratification (30).

## CONCLUSIONS

This study provides the first comprehensive assessment of the predictive value of T2WI and ADC in mpMRI for the pathological grading of prostate cancer through a systematic literature review and meta-analysis. The findings of this meta-analysis support the integration of mpMRI into the diagnostic workflow for prostate cancer. Its improved accuracy could lead to more precise targeting of biopsies, potentially reducing unnecessary procedures and improving the detection of clinically significant cancers. Although mpMRI shows great promise in improving the diagnosis and grading of prostate cancer, its optimal implementation requires addressing challenges related to standardisation, training and cost-effectiveness.
